# High-resolution genetic linkage map of European pear (*Pyrus communis*) and QTL fine-mapping of vegetative budbreak time

**DOI:** 10.1186/s12870-018-1386-2

**Published:** 2018-08-31

**Authors:** Gilad Gabay, Yardena Dahan, Yacov Izhaki, Adi Faigenboim, Giora Ben-Ari, Yonatan Elkind, Moshe A. Flaishman

**Affiliations:** 1 Institute of Plant Sciences, Volcani Research Center, Derech Hamacabim 68, P.O. Box 15159, Rishon Lezion, Israel; 2 The Robert H. Smith Institute of Plant Sciences and Genetics in Agriculture, Faculty of Agriculture, Food and Environment, the Hebrew University of Jerusalem, P.O. Box 12, 76100 Rehovot, Israel

**Keywords:** European pear (*Pyrus communis*), Chilling requirement, Vegetative budbreak, Genetic map, GxE interaction, Linkage group, Phenotype plasticity, QTL, GBS

## Abstract

**Background:**

Genomic analysis technologies can promote efficient fruit tree breeding. Genotyping by sequencing (GBS) enables generating efficient data for high-quality genetic map construction and QTL analysis in a relatively accessible way. Furthermore, High-resolution genetic map construction and accurate QTL detection can significantly narrow down the putative candidate genes associated with important plant traits.

**Results:**

We genotyped 162 offspring in the F1 ‘Spadona’ x ‘Harrow Sweet’ pear population using GBS. An additional 21 pear accessions, including the F1 population’s parents, from our germplasm collection were subjected to GBS to examine diverse genetic backgrounds that are associated to agriculturally relevant traits and to enhance the power of SNP calling. A standard SNP calling pipeline identified 206,971 SNPs with Asian pear (‘Suli’) as the reference genome and 148,622 SNPs with the European genome (‘Bartlett’). These results enabled constructing a genetic map, after further stringent SNP filtering, consisting of 2036 markers on 17 linkage groups with a length of 1433 cM and an average marker interval of 0.7 cM. We aligned 1030 scaffolds covering a total size of 165.5 Mbp (29%) of the European pear genome to the 17 linkage groups. For high-resolution QTL analysis covering the whole genome, we used phenotyping for vegetative budbreak time in the F1 population. New QTLs associated to vegetative budbreak time were detected on linkage groups 5, 13 and 15. A major QTL on linkage group 8 and an additional QTL on linkage group 9 were confirmed. Due to the significant genotype-by-environment (GxE) effect, we were able to identify novel interaction QTLs on linkage groups 5, 8, 9 and 17. Phenotype–genotype association analysis in the pear accessions for main genotype effect was conducted to support the QTLs detected in the F1 population. Significant markers were detected on every linkage group to which main genotype effect QTLs were mapped.

**Conclusions:**

This is the first vegetative budbreak study of European pear that makes use of high-resolution genetic mapping. These results provide tools for marker-assisted selection and accurate QTL analysis in pear, and specifically at vegetative budbreak, considering the significant GxE and phenotype-plasticity effects.

**Electronic supplementary material:**

The online version of this article (10.1186/s12870-018-1386-2) contains supplementary material, which is available to authorized users.

## Background

Genomic improvements have recently created great opportunities for generating high numbers of single-nucleotide polymorphisms (SNPs). Genotyping by sequencing (GBS) [1] has enabled generating high-quality SNP data for genome-wide association studies (GWAS), genetic relatedness studies, high-quality genetic map construction and accurate quantitative trait locus (QTL) detection [2]. This high-throughput technology detects SNP markers that are spread at very high density over the whole genome, enabling the identification of genetic variance between closely related genotypes within a family [3], and can be used to estimate genetic relatedness of species and cultivars. The generated genetic maps are valuable for anchoring scaffolds to pseudo-chromosomes when the genome of the species is not yet organized at the chromosome level [4].

Pear (*Pyrus* spp.), family Rosaceae [2], has great economic value and is considered to be one of the world’s most important perennial deciduous fruit trees, with an estimated yearly production of ~ 26 million tons. The pear genome consists of 17 chromosomes and most of the species in the genus *Pyrus* are diploid (2n = 34), including *Pyrus communis*. The pear genome has been organized to the scaffold level; the draft genome of the Asian pear (‘Suli’) consists of 2103 scaffolds, and the European pear (‘Bartlett’) consists of 142,089 scaffolds covering 577.3 Mb of the estimated 600 Mb genome size [5] and anchors a total scaffold size of 171.4 Mb (29.7%) to the 17 linkage groups (LGs). Recently, a GBS-based genetic map integrated with other available pear genetic maps enabled improving the match between the European pear scaffolds and their LGs and anchoring 50.5% of the genome size. However, to date, a large proportion of the genome scaffolds are unplaced due to short scaffold sequences and lack of high-resolution genetic maps [6].

In recent years, bud dormancy has been studied in perennial trees in the context of climate change [7–9]. The dormant stage plays a major role in the yearly cycle of fruit trees in temperate zones. Active growth following dormancy requires a certain number of chilling hours (chilling units; CUs) during the dormant period [10, 11]. When the chilling requirement (CR) is fulfilled, vegetative budbreak (VB) can occur. Failure to satisfy the CR in a warm winter climate can lead to disorders in VB. The recent rise in temperature worldwide has led to a reduction in CU accumulation. In addition, climate models predict continued global warming, which will also influence CU accumulation [12]. Therefore, fruit tree cultivars with a low CR are needed to deal with the climate change [13, 14], and there is a growing demand for improved pear germplasms to provide breeders with the genetic background to adapt to the climate changes [6, 9]. VB timing has a high heritability value [15] which indicates the potential of breeding for low-CR cultivars. However, the genetic mechanism governing VB date is little understood [16]. In our previous study [17], QTLs associated with VB in European pear were identified on LGs 9 and 8. To the best of our knowledge, this was the only attempt to detect a VB QTL in *Pyrus* spp. In contrast, QTL studies of CR in apple have been conducted [7, 8, 13], with the QTL on LG 9 being the most consistent over years, locations and families. Pear and apple show a high level of synteny [18]. However, there are dissimilarities in marker positions and applicability, and therefore some markers cannot be transferred between the species [19]. Significant improvement in understanding the genetic factors affecting CR was achieved by identifying genes involved in dormancy [20, 21]. Those studies identified six genes, termed *DORMANCY-ASSOCIATED MADS-BOX (DAM)*, in peach [*Prunus persica* (L.) Batsch] and the genomic region termed *evergrowing* (*evg*) locus. These genes have major roles in bud set, vegetative growth regulation and growth cessation [21]. Three *DAM* genes have been identified in pear (*Pyrus* spp.). The first study of transcriptome analysis of bud dormancy in pear (*Pyrus pyrifolia* white pear group) suggested the potential role of DAM genes in pear bud dormancy regulation [22]. Differential expression levels were found in *PpMADS13–1*, a *Pyrus pyrifolia* (Japanese pear) *DAM* homolog gene, during different stages of dormancy [23]. In an earlier study, the other *DAM* genes’ expression levels were correlated with dormancy stages as well (*MADS13–2* and *MADS13–3*) [24]. The CR trait is governed by strong genetic factors; however, its interaction with environmental factors (GxE) has a significant effect on time of VB [17]. GxE in the context of plant breeding is denoted as phenotype plasticity. Breeders aim to produce new cultivars with stable phenotypes in different climatic regions [25, 26]. Selection of new cultivars is usually made in the targeted region. However, climate change can rapidly create different climatic conditions in the same location and therefore cultivars which have phenotype plasticity may perform better with the predicted climate changes. GxE related to important agricultural annual crops has been reported. However, very few studies have been carried out on the GxE effect on fruit tree traits [25].

Breeding fruit trees is a time-consuming process due to limitations such as the juvenile period. These can be reduced by marker-assisted selection that enables selecting genotypes with the required traits at an early stage [27]. The aim of this study was to construct high-resolution genetic maps for better detection and mapping of main genotype effect and GxE QTLs associated with traits of relevance to agriculture in our F1 population, and specifically to enable the development of genomic tools to select low-CR pear genotypes with stable phenotype plasticity over various climatic conditions. We conducted QTL fine-mapping using a high-resolution genetic map covering all 17 pear chromosomes with great density. The methodology, presented in Gabay et al. [17], enabled us to detect the powerful GxE effect and thus carry out GxE QTL analysis. In addition, this study provided additional scaffolds that were not previously anchored to LGs.

## Methods

### GBS

*Pyrus* sp. accessions, F1 progeny and parental cultivar genomic DNA was extracted from young leaves using the DNeasy 96 plant kit (Qiagen, Germany) according to the manufacturer’s instructions. The DNA concentrations were determined by fluorimetry (Qubit, Life Technologies, USA) following the manufacturer’s guidelines. GBS was performed at the Institute of Genomic Diversity, Cornell University; 10% of the samples were examined for quality control: 100 ng of each genomic DNA sample next to 300 ng of digested DNA from the same sample were tested with *Hind*III restriction enzyme on a 1% agarose gel in TAE buffer (Tris base, acetic acid and EDTA) for GBS library preparation as required by the Institute of Genomic Diversity. Because GBS for *Pyrus* sp. is relatively novel, restriction enzyme optimization was required and *Ape*KI restriction enzyme was selected. A total of 162 genotypes out of 180 offspring of the F1 population were subjected to GBS. In addition, the parental cultivars and 19 pear accessions were sequenced. The pear accessions were included to examine diverse genetic backgrounds related to traits with important agricultural impact and to enhance the power of SNP calling. The parental cultivars were sequenced in four replicates to achieve a higher number of reads per site and for accurate SNP calling. All of the sequenced sites with a minimum read depth of 3 per tag were barcoded to compare sites for SNP calling.

### SNP calling and genetic map construction

The first step of SNP calling was performed by the Institute of Genomic Diversity in Tassel 3.0 following the standard pipeline [28]. All samples were subjected to this step. Sites that had more than 10% missing data and a minor allele frequency < 1% were removed. The sites were then aligned to the available *Pyrus* genomes: the ‘Suli’ genome [29] and ‘Bartlett’ genome [5]. A multidimensional scaling (MDS) plot was created using VCFtools v0.1.12a and PLINK v1.07 to assess the genetic relatedness of the F1 population and the pear accessions based on the rate of common filtered SNP alleles between the genotyped samples.

The second step of SNP calling was done separately for the F1 population and the population of pear accessions. The first SNP calling for construction of the F1 population genetic map was carried out by in-house PERL script, discarding homozygous SNPs in both parents. Hence, hhxkk, hhxhh segregation types in the parents were removed. In addition, SNPs with a missing data frequency > 5% and with less than 5 reads per site were discarded. Markers were given code names based on their physical position. SNPs were transformed from fasta files to JoinMap 4.1 files and the adequate markers for genetic map construction were given serial numbers. Three types of segregation were obtained: heterozygous loci in the first parent and homozygous in the second (lmxll), vice versa (nnxnp), and heterozygous loci in both parents (hkxhk). The last stage of SNP calling for the F1 population was carried out by JoinMap 4.1 [30]. Markers with a similarity level of 1 and SNPs that segregated unequally relative to Mendelian segregation (chi-square test, X^2^ < 0.005) were excluded. Map construction was performed by JoinMap 4.1. Genetic distances between markers were calculated using regression mapping strategy with the Kosambi mapping function for CP (cross pollinators, outbreeder full-sib family) population type [31]. A logarithm of odds (LOD) score of 10 was used as the grouping threshold with recombination frequency smaller than 0.4. Another 14 simple-sequence repeat (SSR) markers located on LG 8 and LG 9 were added prior to map construction to evaluate the exact position of the QTLs previously described in Gabay et al. [17]. LG numbers were determined by comparing scaffold numbers to their LGs according to the ‘Suli’ [32] and ‘Bartlett’ [6] maps and are presented in Additional file 1: Table S1. SNP calling for the population of 21 pear accessions was carried out by Tassel 5.0. SNPs with > 5% missing data and with less than the minimal minor allele frequency of 0.05 were excluded.

### Scaffold correlation to European pear genome (‘Bartlett’) LGs

Scaffolds of the set of SNPs that were included in the genetic map of SPD x HS were examined for multiple assignment to their LGs using JMP 13 software. If a scaffold was assigned to more than one LG, all of the scaffold’s SNPs were discarded from the final set of SNPs that was used to construct the genetic maps. To examine the size of the genome coverage by the scaffolds that are presented in the genetic map in this study, we summed the length of the scaffolds in Mbp and calculated the percentage of genome coverage based on the available scaffold sequences of the ‘Bartlett’ v1.0 genome (https://www.rosaceae.org/species/pyrus/pyrus_communis/genome_v1.0. Accessed 10 Nov 2017). We also calculated the additional improvement of the genome coverage compared to recent advances in anchoring scaffolds to pseudo-chromosomes [6] based on the data available in that paper.

### Plant material and phenotyping

The population for this study consisted of 180 offspring derived from a cross between the low-CR cv. SPD and the high-CR cv. HS, for which the phenotyping of VB time was previously described [17]. However, due to limited number of samples that we were able to genotype, we selected randomly 162 offspring to sequence. In addition, 19 *Pyrus* sp. accessions were studied and their phenotyping for VB time is presented here. These accessions were selected from our pear collection at the Agricultural Research Organization’s Volcani Center based on their CRs, i.e., those that had either very low or very high CRs, to examine the genetic variance between groups of significantly different CR accessions. The full list of accessions used in this study is presented in Table 1. Including the parents of the F1 population, 21 accessions were used to identify genetic differences between low-CR and high-CR pear accessions. The aim of this analysis was to support genetic regions that were detected in the F1 SPD x HS population, providing a broader view of the genetic background governing the trait. Four replicates of the same genotypes were exposed to locations with significant differences in CU accumulation during the winter: two replicates in Bet Dagan (BD, low CU accumulation) and two in Tzuba (TZU, high CU accumulation), in 2 consecutive years (2014 and 2015). The TZU replicates were transferred, for a fixed period of time during which most of the CUs are accumulated (1 November until 1 March), from Bet Dagan to Tzuba and then back to Bet Dagan to examine VB time under the same heat conditions after accumulating different amounts of CUs. Evaluations were performed in Bet Dagan under the same conditions for both treatments (BD and TZU), from 1 March until VB date. Climate conditions were collected by the Israeli Meteorological Service and are reported in our previous study [17].

**Table 1:** Days to vegetative budbreak in pear cultivars in two locations over two consecutive years (2014–2015)

		Location				
Cultivar	Species	Bet Dagan		Tzuba		Chilling requirement^a^
Gorham	*P. communis*	135.25	A^b^	95.75	A	High
Beurre Hardy	*P. communis*	134	A	93.5	A	High
Harrow Sweet	*P. communis*	115.5	B	85.5	B	High
Moonglow	*P. communis*	115.25	B	86.25	B	High
Yali	*P. bretschneideri*	115.25	B	83.5	B	High
Highland	*P. communis*	114.75	B	84.5	B	High
Magness	*P. communis*	114	B	85.5	B	High
Abate Fetel	*P. communis*	112.75	B	81.5	B	High
Cascade	*P. communis*	112.5	B	85.5	B	High
Red Clapp	*P. communis*	111.5	B	83	B	High
Bosc	*P. communis*	110.75	B	83.25	B	High
37–6	*P. communis*	102.5	C	71.5	CD	Low
Etruska	*P. communis*	102	C	74.25	C	Low
Lawson	*P. communis*	101.25	CD	75	C	Low
36–7	*P. communis*	99	CD	72	CD	Low
Spadona	*P. communis*	98.5	CD	70.5	CD	Low
6-Jan	*P. communis*	97.75	CD	71.5	CD	Low
Bon Rouge	*P. communis*	95.25	D	73	C	Low
Coscia	*P. communis*	82.75	E	70	CD	Low
Gentile	*P. communis*	81.25	E	67.75	DE	Low
Florida Home	*P. communis x P. pyrifolia*	67.5	F	63.75	E	Low

^a^High chilling requirement > 800 chilling units. Low chilling requirement < 400 chilling units

^b^Levels in a row followed by different letters are significantly different based on comparison by Student’s t-test

### Evaluation of main genotype and GxE effects

To evaluate the VB date in different locations that differ in their variance, normalization was performed according to the following model:

1 $$ {Z}_{ijk}=\frac{{\overline{X}}_{ijk}-{\mu}_{jk}}{\sigma_{jk}} $$

where *Z*_*ijk*_ is the normalized score per genotype *i* in year *j* at location *k*, *μ*_*jk*_ is the mean of the population in year *j* at location *k*, *X*_*ijk*_ is the raw score of genotype *i* in year *j* at location *k*, and *σ*_*jk*_ is the standard deviation of the population in year *j* at location *k*. Statistical analyses were performed with JMP® 13 software (SAS Institute Inc. 2016, JMP® 13 *Profilers*, USA). The values for the VB date trait of the QTL analysis for location and main effect (genotype) were obtained by a mixed linear model (MLM, REML) that evaluates the significance of the effects and the variance component of the factors for the VB date trait based on the following formula:

2 $$ {\mathrm{P}}_{\mathrm{ijkl}}=\upmu +{\mathrm{G}}_{\mathrm{j}}+{\mathrm{Y}}_{\mathrm{k}}+{\uplambda}_{\mathrm{l}}+{\mathrm{G}}_{\mathrm{j}}{\mathrm{Y}}_{\mathrm{k}}+{\mathrm{G}}_{\mathrm{j}}{\uplambda}_{\mathrm{l}}+{\mathrm{Y}}_{\mathrm{k}}{\uplambda}_{\mathrm{l}}+{\mathrm{G}}_{\mathrm{j}}{\mathrm{Y}}_{\mathrm{k}}{\uplambda}_{\mathrm{l}}+{\upvarepsilon}_{\mathrm{ijkl}} $$

The estimate for the GxE interaction, which reflects the differences in genotypes’ VB date between BD and TZU, was obtained according to the following formula:

3 $$ {GxE}_{ij=}{Z}_{ij BD-}{Z}_{ij TZU\kern0.5em } $$

where *GxE*_*ij*_ is the mean interaction value between BD and TZU over 2 years per genotype *i*. *Z*_*ijBD* _is the mean normalized score over 2 years of genotype *i* in BD and *Z*_*ijTZU*_ is the mean normalized score over 2 years of genotype *i* in TZU. Hence, genotypes with *GxE*_*ij*_ values equal or close to 0 showed better stability across locations.

Broad-sense heritability (H^2^) of VB date accounting GxE effects was estimated using the following formula:4where  is the genotypic variance,  is the variance of the GxE interaction (Genotype x Year, Genotype x Location and Genotype x Year x Location),  is the residual error variance estimated from the selected model, *n* is the number of replicates per genotype, and *a* is the number of environments.

### QTL analysis of VB time

QTL analysis was performed by MapQTL 6 [33]. The genetic map that was constructed in this study (SPD x HS) was used to map QTLs. Based on our previous study [17], we showed the significance of the genotype effect and its GxE effects and we therefore carried out four-trait data analysis with BD representing a warm-weather climate, and TZU a cold-climate region. These analysis types used mean normalized values of data recorded over 2 consecutive years (2014 and 2015) in these locations, calculated based on Eq. (1). The QTL for the overall mean (AVG) is the mean value of both locations and the normalized ls means was estimated according to Eq. (2). The QTL for interaction (GxE), which was calculated based on Eq. (3), represents the interaction of the genotypes with two environmental conditions. Automatic cofactor selection was carried out prior to the interval-mapping analysis. When multiple QTLs were obtained, multiple-QTL model (MQM) analysis was conducted. The LOD threshold for QTL significance was obtained by 1000 permutation tests (*P* < 0.05). On LGs where more than one QTL peak exceeded the LOD threshold, the highest peak was declared the QTL. QTL intervals were determined by a 1–2 LOD drop. QTL visualization was performed with Circos [34]. For GxE QTLs, a reaction norm was plotted using JMP 13 software. GxE QTLs were analyzed with both MapQTL 6 and the R software GWAF package [35].

### *Pyrus* sp. accession relatedness and examination of QTLs on diverse genetic backgrounds

The aim of this analysis was to support the QTL regions that were detected in the F1 SPD x HS population and to obtain a broader view of the genetic background governing the trait. Relatedness analysis between the 21 accessions was performed with Tassel 5.0 [36] using the cladogram function based on a neighbor-joining algorithm, which estimates the relatedness based on common alleles. A relatedness tree was plotted with Archaeopteryx software [37]. To support the QTLs associated with VB that were detected in the F1 population in the accession population structure, MLM analysis was conducted with Tassel 5.0 [38]. Significance threshold was obtained by permutation test (*P* < 0.05).

## Results

### SNP calling and genetic map construction

**Fig. 1:**
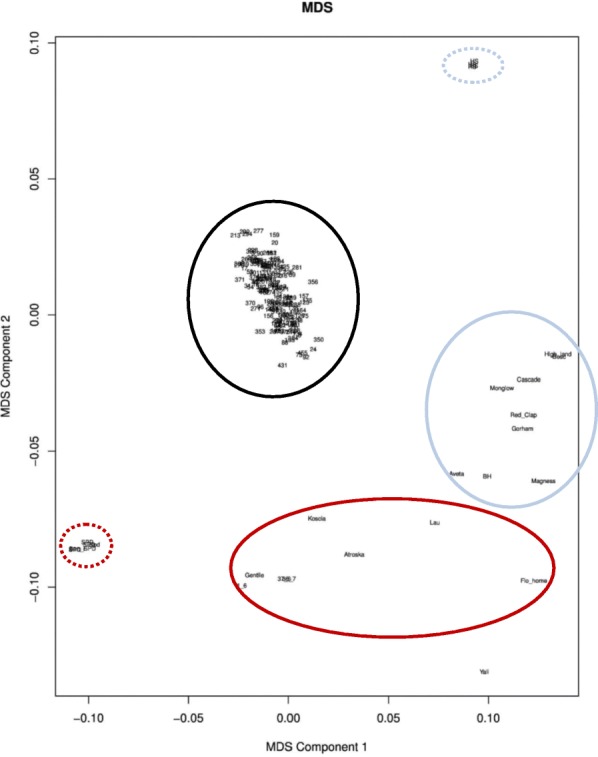
Multidimensional scaling (MDS) of genome-wide SNPs generated by the genotyping-by-sequencing (GBS) pipeline. Low-chilling requirement (CR) cultivars are in red circles. High-CR cultivars are in blue circles. Parents of the F1 SPD x HS population are in red-dotted (SPD) and blue-dotted (HS) circles. F1 SPD x HS are in black circles

The GBS generated a total of 222 million reads with an average of 1.21 million reads per sample; 206,971 SNPs were detected in the *Pyrus× bretschneideri* genome (Asian pear) and 148,622 in the *P. communis* genome (European pear). The mean site depth was 6.76. All samples were included in this step to enhance the power of SNP calling on different genetic backgrounds. The MDS plot (Fig. 1) indicated four clusters: replicates of SPD (parent), replicates of HS (parent), F1 population offspring and two pear accession clusters.

**Fig. 2:**
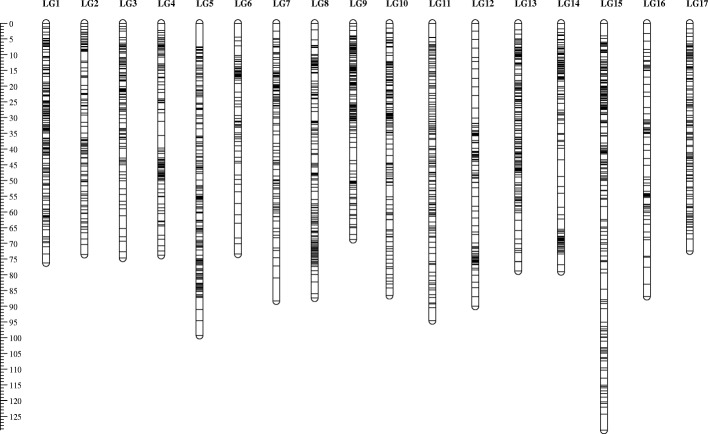
*Pyrus communis* genetic map (SPD x HS). Each number indicates the linkage groups representing the total number of chromosomes of the pear genome (*n* = 17). Black bars indicate SNP marker. The left ruler indicates the length in cM

Further filtering was performed separately on the F1 population and the 21 pear accessions. We considered 16,348 SNPs for genetic map construction of the SPD x HS population, after discarding sites with over 5% missing data and homozygous SNPs in each parent resulting in the same heterozygous allele combination in the whole population. SNPs that were segregated unequally according to Mendelian segregation (X^2^ < 0.005) and markers with a similarity level of 100% were removed prior to genetic mapping. Another 14 SSR markers located on LG 8 and LG 9 were added before map construction to evaluate the exact position of the QTLs that were previously described in Gabay et al. [17]. We obtained 17 LGs representing the chromosomes of the *Pyrus* genome with LOD > 10 for grouping threshold. The map consisted of 2036 markers with length of 1433 cM and an average marker interval of 0.7 cM covering the whole pear genome’s chromosomes with high density (Fig. 2). The full list of LGs and their marker data is given in Additional file 1: Table S1. LG numbers were determined by comparing scaffold numbers to their LGs according to the ‘Suli’ map [32]. The number of markers in these LGs ranged from 68 for LG 6 to 168 for LG 15 (Table 2). The longest LG was 129.4 cM (LG 15) and the shortest was 68.8 cM (LG 9). The LG with the highest average marker distance was LG 16 (1.2 cM), and LG 9 was the most densely covered with 0.5 cM average marker distance. We obtained 36, 097 SNPs for the 21 pear accessions after specific filtering for these accessions.

**Table 2:** Distribution of markers generated by genotyping by sequencing on linkage groups (LGs) of the constructed genetic map (SPD x HS)

LG^a^	Number of markers	Length (cM)	Average marker distance
1	149	76.2	0.5
2	96	73.5	0.8
3	104	74.7	0.7
4	104	73.8	0.7
5	152	99.3	0.7
6	68	73.4	1.1
7	104	88.3	0.8
8	139	87.4	0.6
9	152	68.8	0.5
10	124	86.6	0.7
11	124	94.6	0.8
12	96	90.0	0.9
13	150	78.8	0.5
14	118	79.0	0.7
15	168	129.4	0.8
16	73	86.9	1.2
17	115	72.4	0.6
Total	2036	1433.1	0.7

^a^Linkage groups represent chromosomes of the pear genome

### Scaffold correlation to European pear genome (‘Bartlett’)

**Fig. 3:**
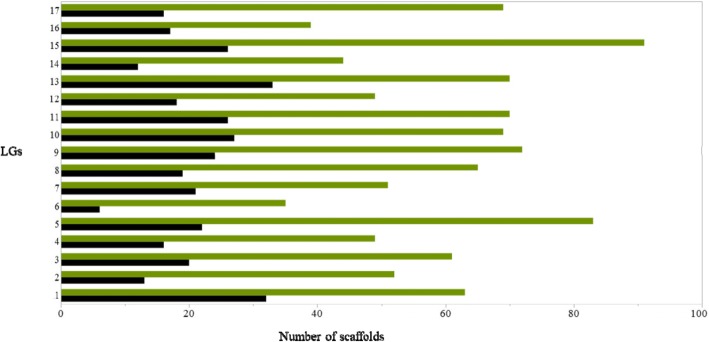
European pear scaffold [5] alignment to the pear genome linkage groups (LGs). The Y axis indicates the LG. The X axis indicates the number of scaffolds. Green bar indicates the total number of scaffolds aligned to the LG. Black bars indicate the new scaffolds aligned to the LGs that were identified in this study

A total of 1030 scaffolds covering a total size of 165.5 Mbp (29%) of the European pear genome (577 Mbp) were aligned successfully to their LGs. Scaffold alignment was compared to the recently published scaffold anchoring to pseudo-chromosomes [6]: 90% (613 scaffolds) of the scaffold alignments to the LGs matched, whereas 10% (69 scaffolds) did not (Additional file 1: Table S2). In addition, we were able to align 348 new, previously unplaced scaffolds covering a total size of 15.4 Mbp of the European pear genome (Fig. 3).

### GxE phenotypic value estimation

**Fig. 4:**
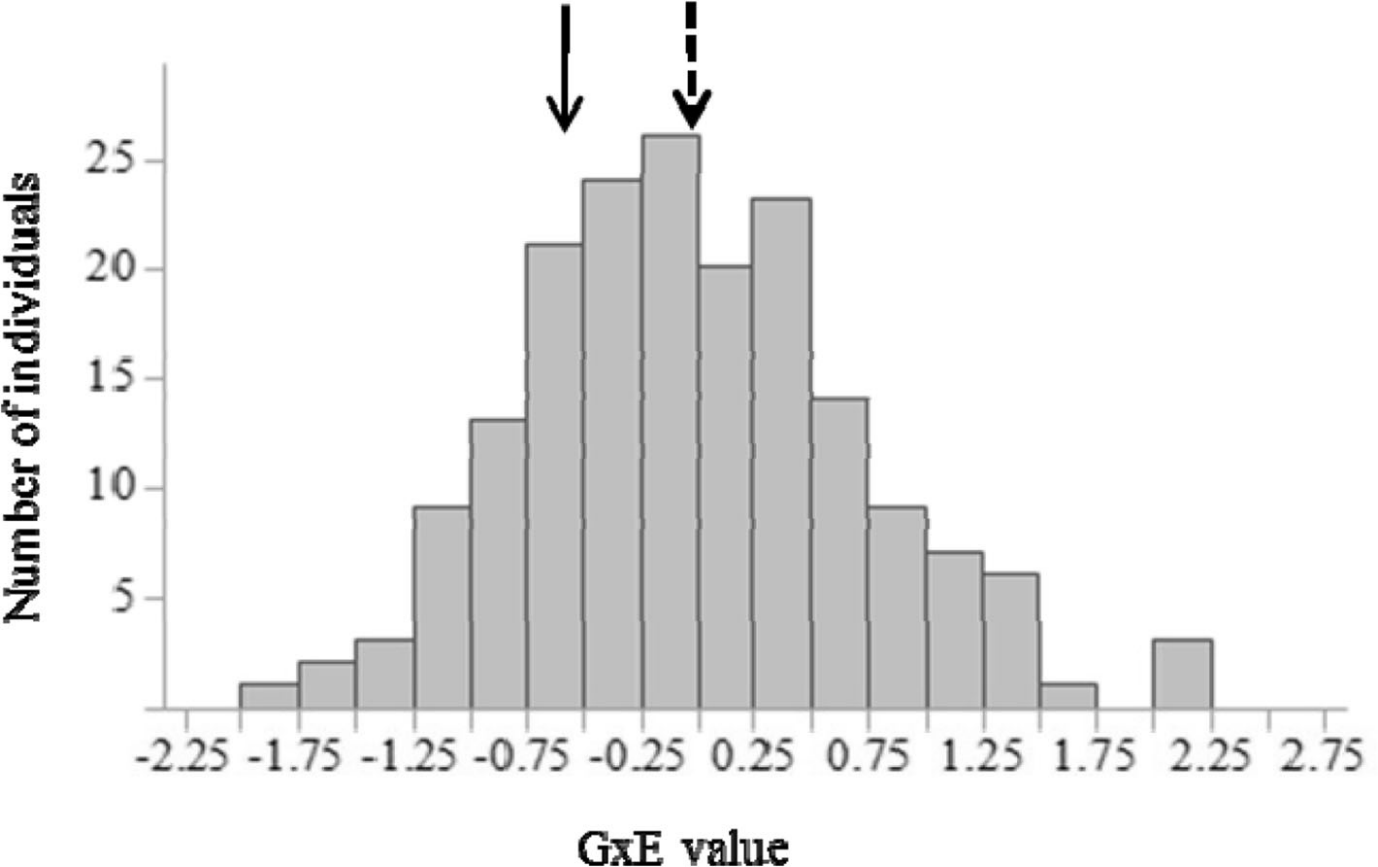
SPD x HS F1 genotype distribution of GxE values calculated based on Eq. (3). Population mean = 0.02, SD = 0.74. Dotted arrow indicates SPD GxE value (− 0.02). Filled arrow indicates HS GxE value (− 0.54)

The distribution of GxE values for the SPD x HS F1 population, consisting of 180 offspring, is shown in Fig. 4. The population mean was 0.02 with SD = 0.74. Extreme genotypes showed unstable phenotypes between BD, a hot climate region with very few CUs (average CUs in 2014–2015 = 187.5) during the winter and TZU, with high CU accumulation (average CUs in 2014–2015 = 702.3). Genotypes with values equal or close to 0 showed better stability across locations. Correlation analysis of the four types of analysis, AVG (overall mean), BD (low CU accumulation), TZU (high CU accumulation) and GxE (interaction between BD and TZU), showed a significant correlation according to Pearson’s test (*P* < 0.0001) among all data types (Table 3), except for the correlation between AVG and GxE values (*P* = 0.21). Hence, there was no significant correlation between the genotypes’ overall mean VB date and their phenotype stability between locations. GxE explained 34.2% of VB date variance and was found significant (*P* < 0.05). The broad-sense heritability (H^2^) estimations for BD, TZU and AVG were 0.66, 0.62 and 0.46, respectively [17]. The H^2^ of the VB date accounting GxE effects according to Eq. (4) was 0.6. The accession population’s GxE effect of Genotype x Location was not significant (*P* > 0.05) and therefore this population was only examined to support QTLs of the main genotype effect, which was significant (*P =* 0.002), and not for GxE QTLs.

**Table 3:** Correlation coefficient matrix for genotype main effect for vegetative budbreak (VB) date; association with location means and GxE values

	BD^a^	TZU^b^	GxE^c^	AVG^d^
BD	1	**0.6**	**0.51**	**0.9**
TZU		**1**	**0.38**	**0.89**
GxE			**1**	0.09^e^
AVG				**1**

^a^Bet Dagan (low chilling units) normalized mean over 2 consecutive years (2014–2015)

^b^Tzuba (high chilling units) normalized mean over 2 consecutive years (2014–2015)

^c^GxE values, the difference in normalized mean between BD and TZU

^d^Overall normalized mean for both locations over 2 consecutive years (2014–2015)

^e^Correlation was not significant (0.21)

Correlation probabilities lower than 0.001 (Pearson’s test) are in bold

### Phenotypic trait assessment

The VB time phenotypes of 180 F1 offspring (SPD x HS) and their parents have been previously described [17]. Results of the phenotyping of an additional 19 pear accessions are presented here for the first time. CU accumulation is known to have an impact on VB date and its variance. Hence, VB date of the individuals that were subjected to the colder climate in TZU was earlier than that of individuals exposed to the warmer climate in BD (Table 1). The distribution of the normalized mean VB date of the accessions per location and with regard to the F1 population is shown in Additional file 2: Figure S1. The accessions were selected according to major differences in their CRs to estimate genetic variance between low-CR and high-CR cultivars; therefore, the accessions’ VB dates were either very early or very late, representing low-CR and high-CR cultivars, respectively (Additional file 2: Figure S1).

### QTL analysis of VB time (main effect) and its interaction with location (GxE)

**Fig. 5:**
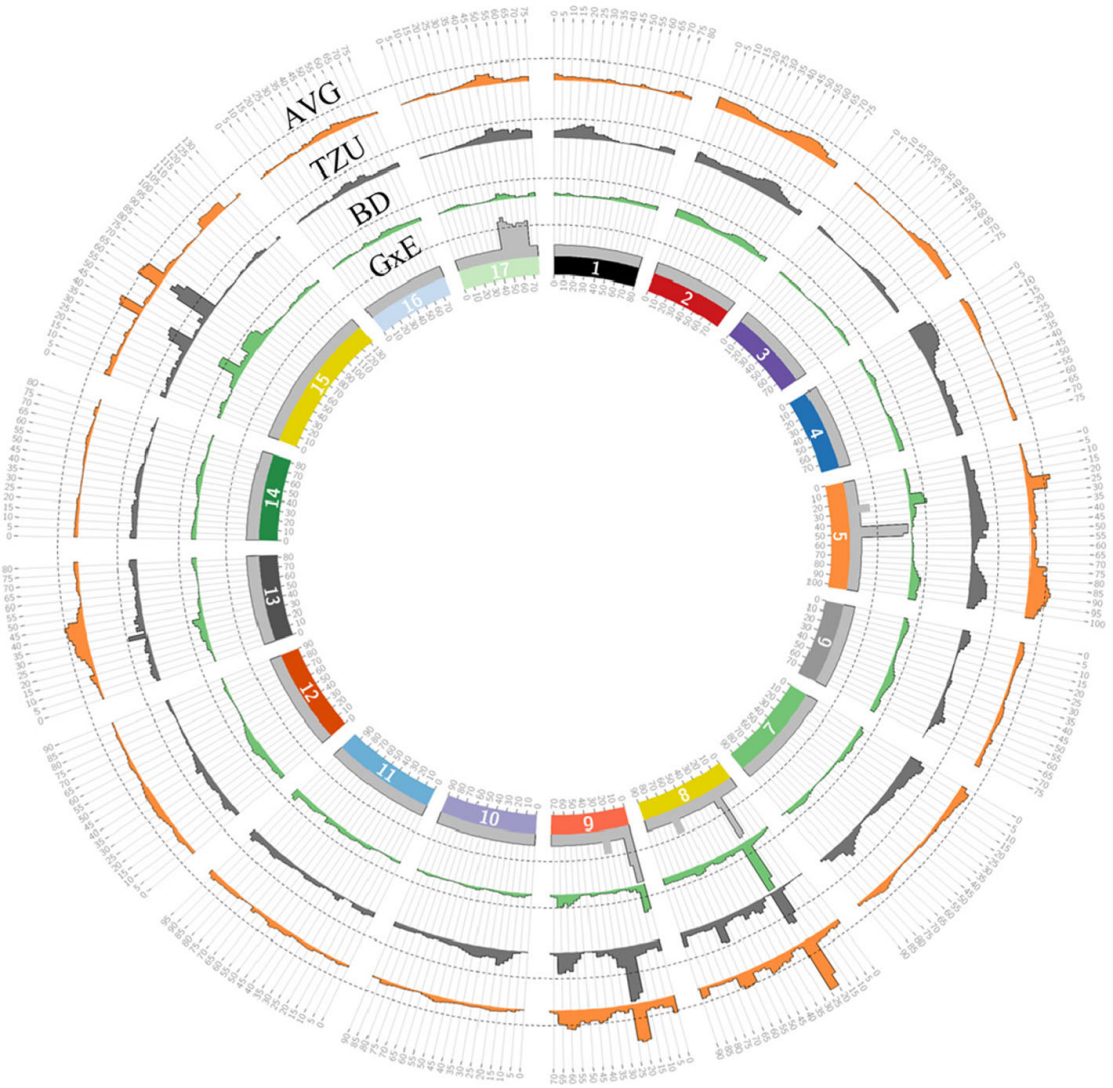
QTL positions for main genotypic and GxE effects on VB time. Each circle indicates the type of analysis. Overall mean (AVG) = orange, Tzuba (TZU) = gray, Bet Dagan (BD) = green, interaction (GxE) = light gray. Linkage groups (LGs) are indicated by numbers on the inner ring. Dotted lines indicate LOD thresholds determined by permutation test

Significant QTLs for pear VB were detected in both warm (BD) and cold (TZU) climates along with overall mean (AVG) QTLs (Fig. 5). New AVG QTLs were detected on LGs 5, 13 and 15 and the QTLs on LGs 8 and 9 were confirmed. All of the GxE QTLs were identified for the first time in this study. In BD, a major QTL was detected on LG 8; the LOD score of the QTL peak was 14.72 and it explained 34.4% of the phenotypic variance. Additional minor QTLs were detected on LGs 9, 15 and 5, explaining 9.1%, 7.9% and 5% of the variance, respectively (Table 4). In TZU, the most significant QTL was identified on LG 9 (LOD = 8.05), explaining 10.6% of the variance. Other QTLs were found on LGs 8 (R^2^ = 7.5%) and 15 (R^2^ = 9.1%). A major QTL for the overall mean (AVG), revealing genotype means of two replicates over 2 years (2014 and 2015) in both locations, was detected on LG 8. The QTL peak’s LOD was 11.49, and 28% of the phenotypic variance was explained by this QTL. Additional QTLs were found on LGs 5 (R^2^ = 5.2%), 9 (R^2^ = 9.8%), 13 (R^2^ = 4.2%) and 15 (R^2^ = 6%). Significant QTLs for GxE interaction were detected on LGs 5 (R^2^ = 10.3%) and 9 (R^2^ = 10.9%). Additional GxE QTLs were found on LGs 8 and 17 (Table 4). For each GxE QTL, a reaction norm was plotted to examine the mean phenotypic value for any allele combination in BD and TZU of the markers located at the GxE QTL peak (Additional file 2: Figure S2). Similar QTLs were detected by R software, GWAF package (data not shown). On LGs 5, 8 and 9, the same genotype variances were ranked differently between TZU and BD. Hence, the same genotypes acted differently under different environmental conditions. On LG 17, no rank cross between any of the allele combinations was detected; however, the mean phenotype value was higher in TZU for all allelic combinations (Additional file 2: Figure S2).

**Table 4:** MQM analysis of vegetative budbreak (VB) date in F1 SPD x HS population for locations with different climate conditions, genotype main effect and GxE

Analysis type	LOD threshold (GW)^a^	LG^b^	LOD score	QTL peak position (cM)^c^	% Variance^d^
Bet Dagan	3.5	5	4.1	26.3	5.0
		8	14.7	22.4	34.4
		9	7.0	1.0	9.1
		15	6.1	38.7	7.9
Tzuba	3.5	8	5.5	21.4	7.5
		9	8.1	22.3	10.6
		15	7.0	62.9	9.1
AVG	3.5	5	4.5	21.9	5.2
		8	11.5	21.4	28
		9	7.8	19.5	9.8
		13	3.9	42.8	4.2
		15	4.9	40.7	6.0
GxE	3.8	5	5.2	43.6	10.3
		8	5.0	17.3	9.7
		9	5.5	1.0	10.9
		17	3.9	44.5	6.8

^a^Genome-wide LOD threshold obtained by 1000 permutation test

^b^Linkage group

^c^Position of the highest LOD score within the LG

^d^Percentage of VB date variance explained by the QTL

### *Pyrus* sp. accession relatedness

**Fig. 6:**
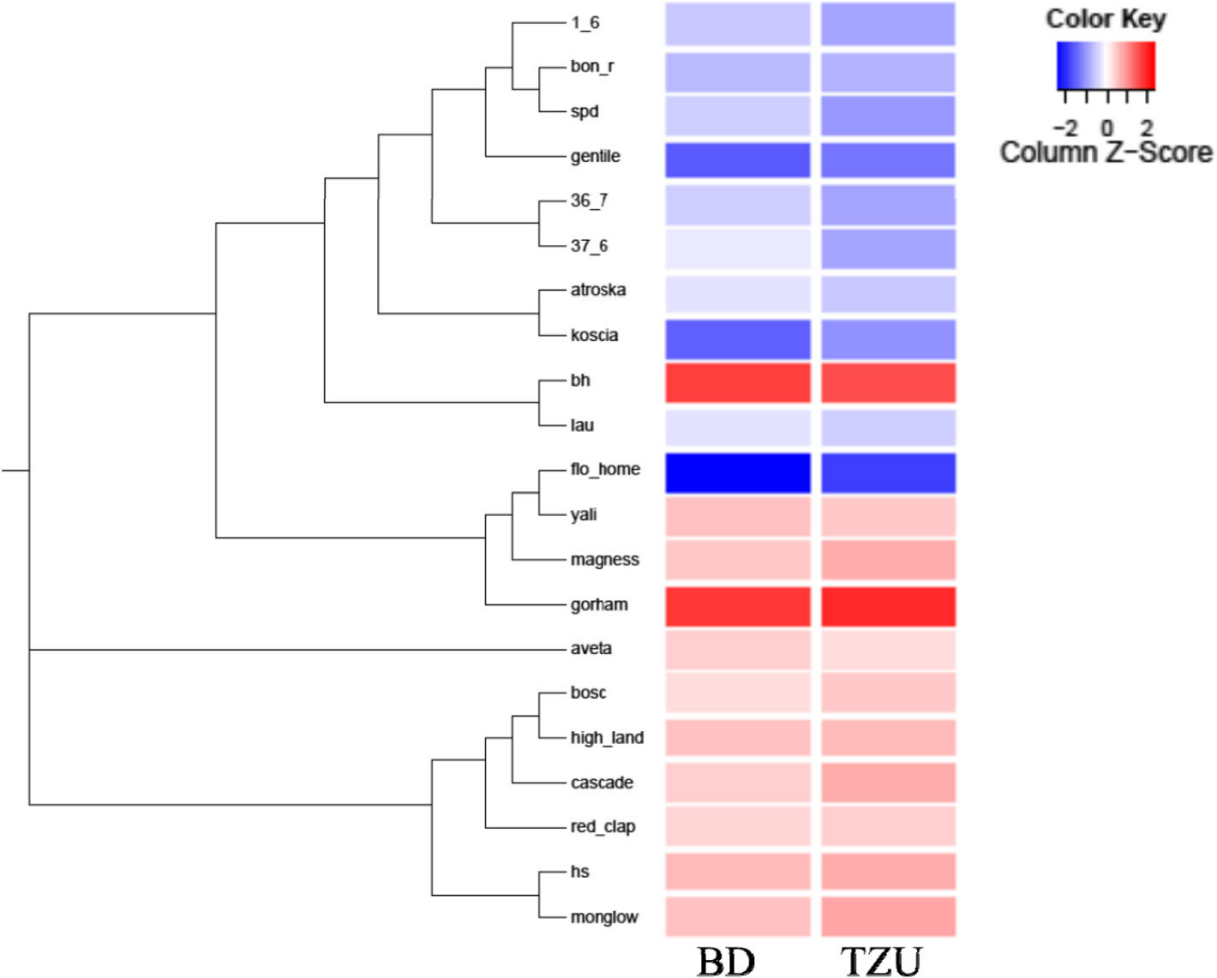
Relatedness tree of 21 *Pyrus* sp. accessions performed with cladogram function based on neighbor-joining algorithm. VB normalized date is indicated in the color key, i.e., − 2 indicates early VB date and 2 indicates late VB date

The accession relatedness analysis was conducted with Tassel 5.0 [36] using the cladogram function based on the neighbor-joining algorithm. We tested 36,097 SNPs to calculate the distance between each pair of accessions. A relatedness tree, indicating the distance between the various accessions, is shown in Fig. 6 based on the matrix distance generated by Tassel 5.0. A distance of 0 represents the same genotype whereas a distance of 1 represents no common allele between the different accessions. The parents of the F1 population, SPD and HS, had the highest relatedness distance value (0.367; Additional file 1: Table S3). Hence, these cultivars have the least number of common SNP alleles among the screened accessions. The lowest relatedness distance value was found between ‘Bosc’ and ‘High Land’ (0.15), representing the highest genetic similarity within this population (Additional file 1: Table S3). Low-CR cultivars and high-CR cultivars were located on different branch clusters (Fig. 6), except ‘Florida Home’ and ‘Beurre Hardy’, indicating that there is high genetic similarity within each CR group. These clusters were also identified in the MDS plot (Fig. 1).

### Phenotype–genotype associations for main genotype effect in the pear accessions

The SNPs that were significantly associated to the VB trait are presented in Additional file 1: Table S4. However, some SNPs that were found significant (*P <* 0.05) after permutation test were not mapped successfully to the SPD x HS genetic map due to similarity to other markers, unequal Mendelian segregation, or homozygosity in both parents. Significant markers were found on every LG to which AVG, BD and TZU QTLs were mapped (Table 5). However, not all of these markers were located within the highest peak of the QTL intervals. Segregation of the markers that were found significant in both MLM and single-marker analysis of phenotype–genotype associations in the pear accession population is presented in Additional file 2: Figure S3. The GxE effect was not significant in the accession population analysis and therefore we could not use this population to support the GxE QTLs that were detected in the F1 population.

**Table 5:** Summary table for MLM analysis of 21 pear accessions. Significant markers obtained in MLM analysis and their matching to the main genotype (AVG), Bet Dagan (BD) and Tzuba (TZU) QTLs region of the F1 population (SPD x HS)

LG^a^	Significant markers^b^	QTL interval^c^			Second QTL interval^d^		
		AVG	BD	TZU	AVG	BD	TZU
5	5			*	1		*
8	25	1	1	1	1		
9	5	2	1	2			
13	2		*	*		*	*
15	6				2		

^a^Linkage group

^b^Total number of significant markers obtained by the MLM analysis within the LG

^c^Number of significant markers obtained by the MLM analysis within the main QTL interval

^d^Number of significant markers obtained by the MLM analysis within the second QTL interval. Refers to the second highest peak QTL when two peaks were observed

^*^QTL was not detected in this data analysis

## Discussion

### High-resolution genetic map of European pear

GBS is a useful tool for generating large-scale data toward the construction of high-resolution genetic maps [28]. In pear, there has only been one successful attempt at using the GBS approach [6] for genetic map construction. After strict filtering, GBS was undertaken for all samples to detect SNPs accurately by examining diverse genetic backgrounds, and later separately for the F1 population and pear accessions to generate large amounts of SNP data. We constructed a dense SNP-based high-resolution genetic map to detect QTLs for traits with relevance to pear breeding programs and we were able to identify QTLs associated to VB with small intervals (Fig. 5); this allowed us to detect the relevant genomic regions more accurately in the whole genome. In this study, we used an F1 population (SPD x HS) to genotype 162 offspring and 21 pear cultivars to support the QTLs that were detected in the F1 population. The constructed genetic maps may be useful for other genomic studies in pear, specifically in SPD x HS populations, for a better understanding of the genetic mechanisms governing other important agricultural traits. Today, pear genomes are organized to the scaffold level [5, 29]. GBS enables us to generate genetic maps which can contribute to the European pear genome study [5]. The genetic map data, which link scaffolds of the mapped markers to both European and Asian pear LGs, are provided in Additional file 1: Table S1. In this study, we aligned an additional 348 scaffolds covering 15.4 Mbp of the pear genome.

### QTL fine-mapping of genetic and GxE effects associated to VB date

Several VB-associated QTLs have been identified in closely related species, including *Malus × domestica* Borkh [7, 8, 13] and other Rosaceae members [39]. In this study, the most significant QTL was detected on LG 8 and confirmed the QTL that was detected on the same LG in our previous study [17], with a LOD score of 11.49, explaining 28% of the phenotypic variance. However, in this study we used a high-resolution genetic map and therefore were able to more accurately detect the QTL peak position (21.4 cM on LG 8). The QTL on LG 9 (R^2^ = 9.8%) was detected and confirmed as well (Table 4). Moreover, in this study, use of GBS enabled us to examined QTLs on all LGs and therefore, we detected new main genotype effect QTLs for pear VB on LGs 5, 13 and 15. Furthermore, the QTL analysis, using dense genetic maps that cover the whole genome, enabled us to more accurately estimate the phenotypic variance explained by the QTLs that were detected in the entire genome and not only the specific QTLs that were examined in Gabay et al. [17].

Pear and apple show high levels of synteny [5, 18]; however, although several studies have detected a major QTL on LG 9 [7, 8, 13], in this study, the major AVG QTL was detected on LG 8. The additional QTLs found here on LGs 5, 9, and 15 have been identified in apple [7]. However, a new QTL associated to VB date in both pear and apple was detected on LG 13; to the best of our knowledge, this QTL has never been identified in pear or apple. These dissimilarities between apple and pear emphasize the importance of conducting genetic studies specifically in pear to reveal QTLs and genes governing important traits, even though the two species share a high level of synteny. *PpDAM1* and *PpDAM2,* two of the *DAM* genes that have been identified in *Pyrus* [9, 23, 24], were located on scaffold 293.0, the same scaffold where the flanking markers of the major QTL interval on LG 8 were located, marker 10,980 and marker 10,954. The physical position matches our major QTL interval on LG 8. Differential expression levels have been found for *PpDAM1* and *PpDAM2* at different stages of dormancy [23, 24]. Therefore, we assume that these genes play a major role in regulating the genetic mechanism governing VB.

By exposing replicates of the same genotypes to vastly different numbers of CUs but the same heat conditions during 2 consecutive years, we were able to detect GxE QTLs associated with VB for the first time in fruit trees. Four significant QTLs were detected on LG 5, LG 8, LG 9 and LG 17 (Fig. 5). Those QTLs reflected genotypes carrying alleles that show tremendous differences in VB date between the two locations with different climatic conditions. Hence, the same genotype acts differently in different environments.

### Genotype plasticity with climatic change

In the context of global warming, the main effect QTL for time of VB is needed to select genotypes that are suited to warmer areas. However, although climate change can be predicted, a genotype’s adaptation to the new environment cannot. The importance of adequate VB timing is relevant in both warm and cold regions for low CR, due to frost susceptibility [40]; therefore, both climate regions require stable cultivars with low GxE effects. The general assumption is that when the CR is fulfilled, budbreak will occur under favorable conditions. It was therefore surprising to detect that the normalized VB time of a replicate genotype exposed to more CUs was later than that of other replicates of the same genotype exposed to less CUs. For instance, the normalized VB date of genotype 143 in BD was − 1.48, which was early compared to the rest of the population, and moderately late in TZU (0.46). Phenotypic plasticity reflects the stability of a certain genotype in different climate regions. We assume that stable genotypes, which were scored with GxE values close to 0 according to Eq. (3) and were examined under various conditions, will remain stable under the expected rising temperatures and climate change in coming years. For instance, genotypes 134 and 21 had almost the same AVG phenotype value, − 1.36 and − 1.35, respectively. However, genotype 134 (GxE value = − 0.25) was more stable between locations than genotype 21 (GxE value = − 1.18), and we therefore assume that genotype 134 will be more suitable for adaption to a changing climate.

### Accession population study

To support the QTLs that were detected in the F1 SPD x HS population, we examined 21 pear accessions that differ in their CRs. This population size might not be sufficient for GWAS in and of itself since the differences may result from other genetic variances that are not associated with CR [41]; we therefore used this population to gain supporting information. For all LGs on which a QTL was detected in the F1 population, we found significant markers in the MLM analysis of the pear accessions on the same LG (Table 5). The relatedness tree, which reflects the level of identity between the accessions carrying the same alleles, identified two clusters (Fig. 6) corresponding to CR, indicating that those cultivars may have the same genetic background for the CR trait. An exception from the low-CR group was ‘Florida Home’ that is derived from a cross between European pear and Asian pear [42]; we therefore assume that this extremely low-CR cultivar has a different genetic background for CR determination. No GxE effect was detected in the analysis of the accession population, perhaps due to the selection of extreme cultivars. Hence, the strong main genotype effect masked the GxE effect. In addition, some of those cultivars have been commercially grown for decades and we therefore assume that they were also selected for phenotype stability over years and locations.

## Conclusions

To date, the European pear genome has been organized to the scaffold level [5, 6]. However, demand for the development of genomic tools that will accelerate pear breeding programs calls for more efficient pear genomic resources. In this study, we confirmed 90% of the scaffold alignments in a recent European pear genome study [6] and we were able to add another 348 (15.4 MbP) previously unplaced scaffolds.

Along with detected main genotype (AVG) QTLs, we suggest genomic selection tools that can greatly accelerate the lengthy breeding process to ensure that cultivars will adapt to a changing climate. Furthermore, our use of diverse genetic backgrounds of different pear accessions supports the suggested stability of the QTLs across genetic backgrounds. Today’s climate change makes selecting well-adapted cultivars a great challenge, because selection for a target location cannot ensure good adaption, due to climate variations within that location. Hence, cultivars that are selected today will not be suited for growth in the same location in the future. Our suggested selection strategy considers selection with significant GxE effects. This strategy was designed based on our data (Additional file 2: Figure S4). Hence, a certain genotype should not be selected only for its overall mean phenotypic value but also for its phenotype plasticity after being examined under various climate conditions. This will ensure adequate timing of VB in the climate region from which it was selected as the climate changes.

## Additional files

Additional file 1: **Table S1.** Marker data for the F1 SPD x HS genetic map consisting of 17 linkage groups constructed in this study. **Table S2.** European pear genome Scaffold alignment to linkage groups. **Table S3.** Neighbor-joining matrix of common SNP alleles within the 21 pear accessions. **Table S4.** Association mapping results of significant markers exceeding the permutation test for chilling requirements of 21 Pyrus spp. accessions. (XLS 347 kb)

Additional file 2: **Figure S1.** Mean vegetative budbreak (VB) date and normalized phenotypic value distribution for SPD x HS F1 population and 21 *Pyrus* sp. cultivars with different chilling requirements (CRs). ** Figure S2.** Reaction norm plot for GxE QTLs. Comparison of genotypes’ normalized vegetative budbreak (VB) mean, carrying each of the allele combinations (ac, ad, bc, and bd) between Tzuba (TZU) (high chilling units) and Bet Dagan (BD) (low chilling units). **Figure S3.** Significant SNP markers associated with vegetative budbreak (VB) date, and segregation of low-chilling requirement (CR) cultivars and high-CR cultivars based on MLM and single-marker analysis. (DOC 799 kb)

## Abbreviations

BD: Bet Dagan
CR: Chilling requirement
CU: Chilling unit
DAM: DORMANCY-ASSOCIATED MADS-BOX
GBS: Genotyping by sequencing
GWAS: Genome-wide association studies
GxE: Genotype-by-environment interaction
LG: Linkage ggroup
LOD: Logarithm of odds
MDS: Multidimensional scaling
MLM: Mixed linear model
MQM: Multiple-QTL model
QTL: Quantitative trait locus
SNP: Single-nucleotide polymorphism
SSR: Simple-sequence repeat
TZU: Tzuba
VB: Vegetative budbreak
