# Supplementary material for: High-resolution genetic linkage map of European pear (Pyrus communis) and QTL fine-mapping of vegetative budbreak time
Source: BMC Plant Biol. 2018 Aug 31;18:175. doi: 10.1186/s12870-018-1386-2 (PMC6117884; doi:10.1186/s12870-018-1386-2)
Supplement: Supplementary file 2 — Figure S1. Mean vegetative budbreak (VB) date and normalized phenotypic value distribution for SPD x HS F1 population and 21 Pyrus sp. cultivars with different chilling requirements (CRs). Figure S2. Reaction norm plot for GxE QTLs. Comparison of genotypes’ normalized vegetative budbreak (VB) mean, carrying each of the allele combinations (ac, ad, bc, and bd) between Tzuba (TZU) (high chilling units) and Bet Dagan (BD) (low chilling units). Figure S3. Significant SNP markers associated with vegetative budbreak (VB) date, and segregation of low-chilling requirement (CR) cultivars and high-CR cultivars based on MLM and single-marker analysis. (DOC 799 kb) [file 12870_2018_1386_MOESM2_ESM.doc]

**Figure S1** Mean vegetative budbreak (VB) date and normalized phenotypic value distribution for SPD x HS F1 population and 21 *Pyrus* sp. cultivars with different chilling requirements (CRs). High-CR cultivars are marked in blue. Low-CR cultivars are marked in red. F1 SPD x HS are marked in blank. (a) Genotypes' mean VB date over 2014–2015 for trees exposed to chilling units (CUs) in Bet Dagan (BD). (b) Genotypes' mean normalized phenotypic value over 2014–2015 for trees exposed to CUs in BD. (c) Genotypes' mean VB date over 2014–2015 for trees exposed to CUs in Tzuba (TZU). (d) Genotypes' mean normalized phenotypic value over 2014–2015 for trees exposed to CUs in TZU. Average CU in BD = 187.5, TZU = 702.3. The X axis for (a,c) indicates days from 1 Jan of each year (Day 0 = 1 Jan 2014 or 1 Jan 2015). The X axis for (b,d) indicates normalized phenotypic value according to equation (1)

**Figure S2** Reaction norm plot for GxE QTLs. Comparison of genotypes' normalized vegetative budbreak (VB) mean, carrying each of the allele combinations (ac, ad, bc, and bd) between Tzuba (TZU) (high chilling units) and Bet Dagan (BD) (low chilling units). GxE QTLs on (a) LG 5, (b) LG 8, (c) LG 9, (d) LG 17

**Figure S3** Significant SNP markers associated with vegetative budbreak (VB) date, and segregation of low-chilling requirement (CR) cultivars and high-CR cultivars based on MLM and single-marker analysis. High-CR cultivars are in a blue frame; low-CR cultivars are in a red frame. Letters indicate the SNP nucleotides; A = A:A, C = C:C, G = G:G, T = T:T, R = A:G, Y = C:T, S = C:G, W = A:T, K = G:T, M = A:C, + = +:+ (insertion), 0 = +:-, - = -:- (deletion), N = unknown

**Figure S4** GxE values vs. overall mean of the F1 SPD x HS population genotypes. Genotype differences in normalized scores of vegetative budbreak date between locations with high chilling units (TZ) and low chilling units (BD) and their mean


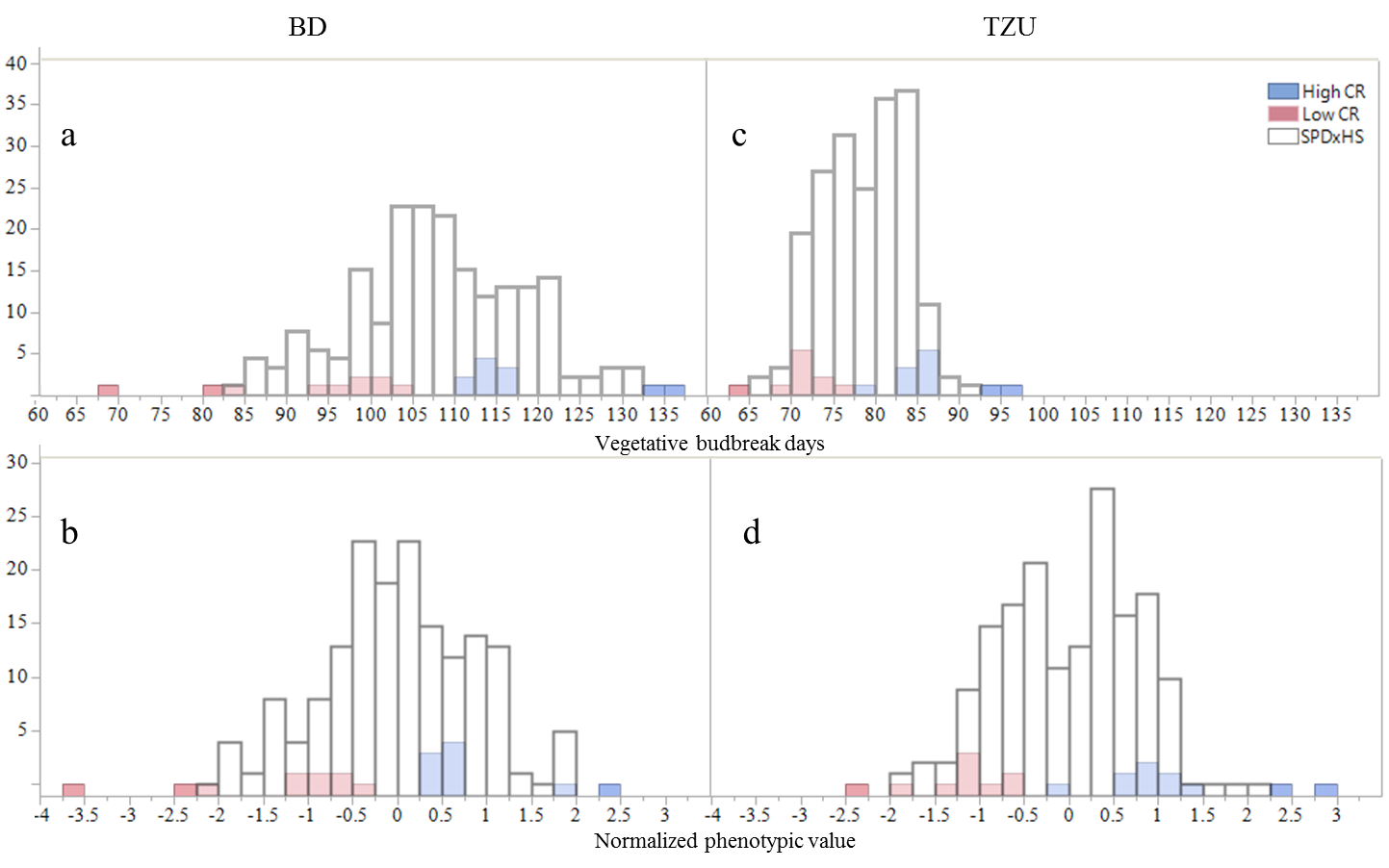


**Figure S1**


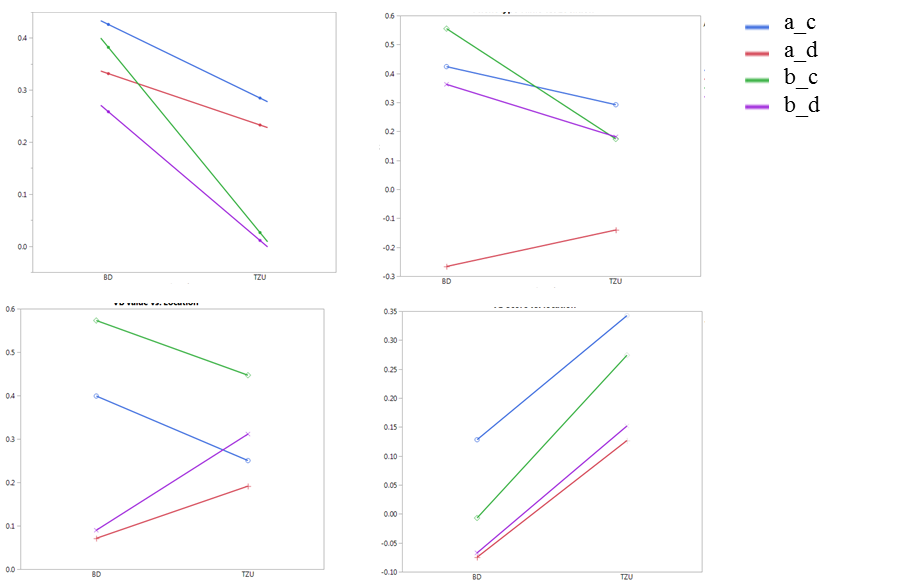


a

b

d

c

**Figure S2**


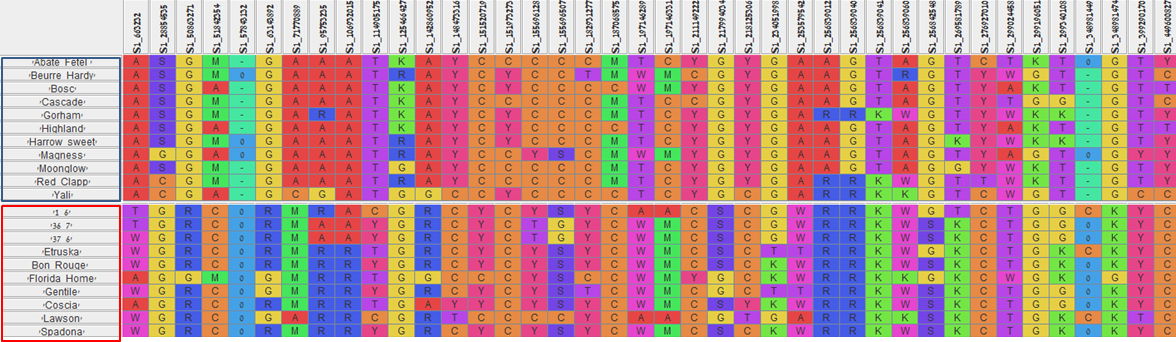


**Figure S3**


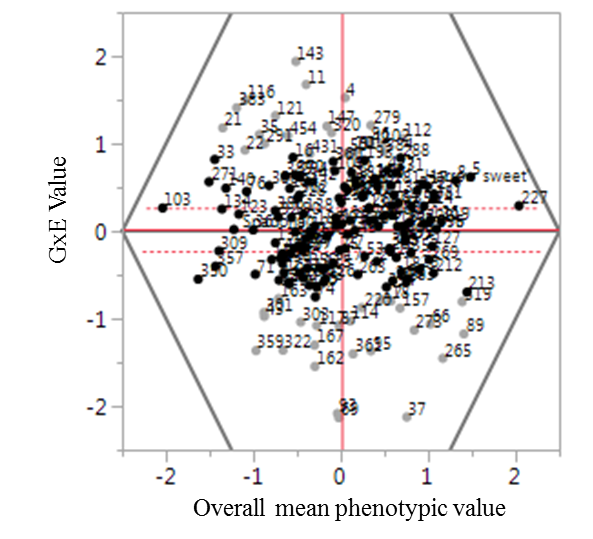


Figure S4
